# Editorial: Advanced microfluidics for synthetic biology and/or biomedical applications

**DOI:** 10.3389/fbioe.2024.1440206

**Published:** 2024-06-28

**Authors:** Xiang Ren, Miao Yu, Inyoung Kim, Wonil Nam, Yuxiang Qin

**Affiliations:** ^1^ Department of Microelectronics, Tianjin University, Tianjin, China; ^2^ Department of Research and Development, Stedical Scientific, Carlsbad, CA, United States; ^3^ Department of Statistics, Virginia Tech, Blacksburg, VA, United States; ^4^ Department of Electronics Engineering, Pukyong National University, Busan, Republic of Korea

**Keywords:** lab on a chip, BioMEMS (biomedical microelectromechanical systems), advanced microfabrication, synthetic biology, artificial intelligence, microfluidics

The advanced microfluidics with multi-modal biosensing technologies have become increasingly popular in the Bio-micro-electro-mechanical system (BioMEMS) field for synthetic biology and/or biomedical applications. The advanced microfluidics technologies include improvements on traditional lab-on-chip (LOC), point-of-care (POC), organ-on-chip, programmable microfluidics, and recent developed lab-at-home (L@H) techniques (advanced BioMEMS technologies compatible with smart devices for daily usage at home).

The Research Topic in this Research Topic “Advanced Microfluidics for Synthetic Biology and Biomedical Applications” covers multiple approaches in advanced microfluidics with novel LOC techniques and artificial intelligence (Gasulla et al. in this Research Topic) with diversified topics, technologies, and applications.

**FIGURE 1:**
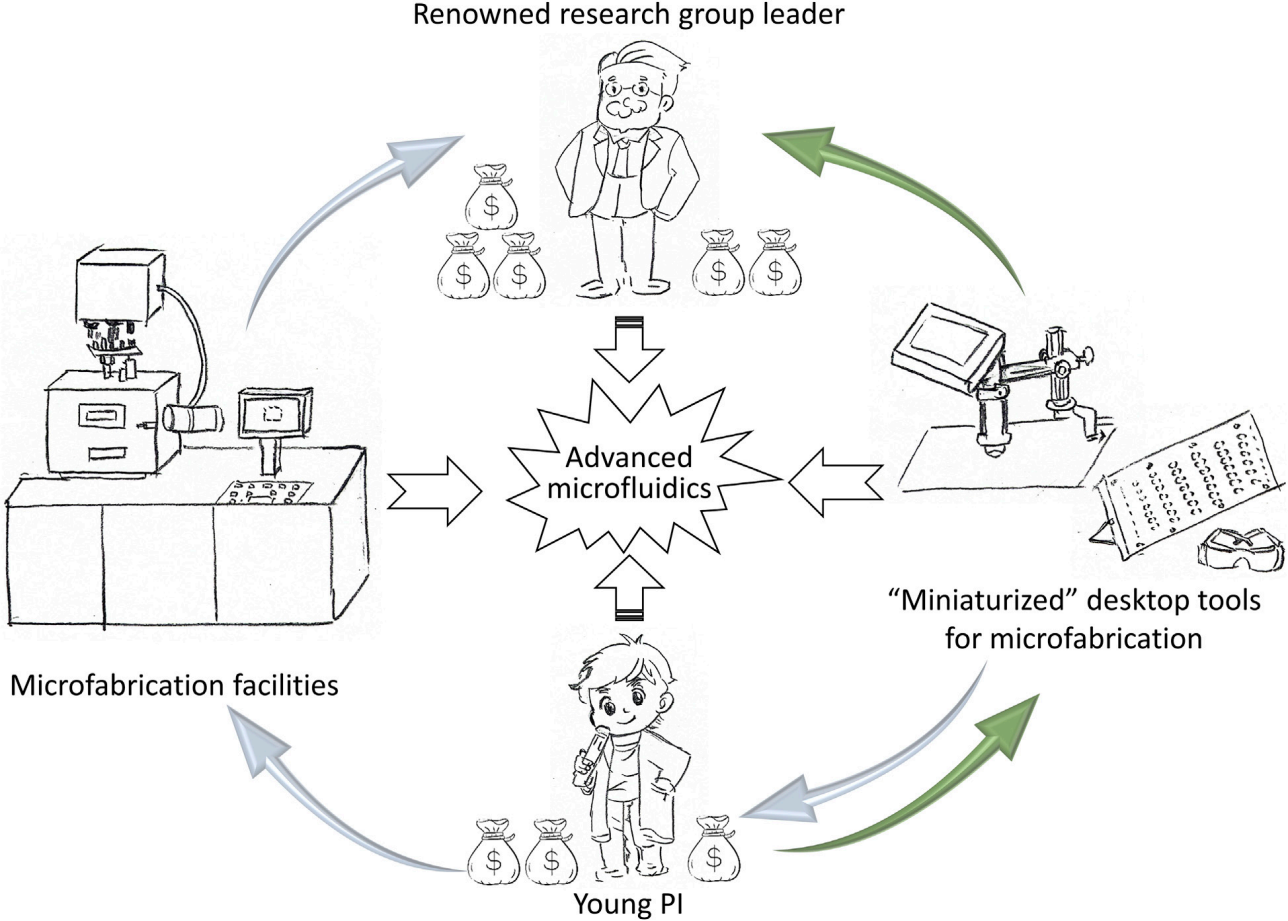
Young PI’s career path on advanced microfluidics.

Due to sophisticated platforms and expensive facilities required, major developments in MEMS techniques are usually created by well-established laboratories. Advanced microfluidics, or advanced MEMS, offers economical options on top of traditional MEMS techniques and brings more opportunities for researchers to establish broader applications. Many young PIs have difficulties in exploring biological or biomedical platforms in MEMS field. Instead of seeking collaborations with well-established wet labs and biological labs, the young PIs tend to explore new methodologies and approaches by themselves (Figure 1). As quoted from “Jurassic Park”: “life will find a way.“, two major directions are applicable for independent young PIs: 1) developing a preliminary research route without the usage of animal cells, and this approach requires the approval from the Institutional Review Boards (IRB) or Institutional Animal Care and Use Committee (IACUC) in MEMS; 2) applying economical tools to MEMS processes and exploring their impacts.

Driven by the strong computing ability and novel tools, partial capabilities of microfabrication (e.g., economical additive manufacturing and stereolithographic tools) are to achieve micro-scale resolution. Taking photolithography as an example, traditional photolithography requires expensive mask aligner and photoresist processes with comprehensive baking protocols. However, people can design less complicated microfluidic configurations using “single-mask” photolithography or UV treatment to achieve one or multi-dimensional microstructures with less expensive UV light sources, such as UV LED arrays. In this Research Topic, Chu et al. designed a simple microfluidic system mimicking tomato xylem; Contato et al. constructed simple microfluidic structures for cell seeding. Those microfluidic systems can be fabricated by “single-mask” desktop photolithography procedures to reduce the cost significantly. Utilizing the additive manufacturing tools for soft-lithography is another application. Huang et al. in this Research Topic used an economical 3D printer for their microfluidic devices. The strategies discussed in this Research Topic provided economical approaches for young PIs to explore research directions.

Advanced microfluidics serves as a key technology in multiple disciplines, including electrical engineering, mechanical engineering, biomedical engineering, chemistry, and biology, etc. This Research Topic presents diversified applications in areas such as COVID-19 Gasulla et al., heart organ on chip Contato et al., cells on chip Huang et al., xylem-mimicking microfluidics and biofilm Chu et al. One of the benefits of conducting multidisciplinary research with advanced microfluidics and BioMEMS is that a young PI can establish a research laboratory within a relatively short time and collaborate with other well-established groups at the same time. For instance, traditional biomedical researches involving “gold standard” protocols for biological substances may not be suitable for biomedical application scenarios, such as polymerase chain reaction (PCR), enzyme-linked immunosorbent assay (ELISA), and Western blot, etc. The scientists continuously develop novel devices or platforms to expand detection limits and challenge the “gold standard” in BioMEMS. Taking PCR as an example, PCR has been serving as a “gold standard” in most nucleic acid detection methods, even though the reliability and the detection limits are criticized by major publications. The standard PCR cannot prevent single base mutations, nor prevent target loss with extremely low concentrations. On the contrary, advanced BioMEMS can eliminate restrictions in PCR and create a platform with high sensitivity sensors/transducers to detect biological substances, which provides young PIs with more options in developing new tools (Figure 1).

The advanced BioMEMS with artificial intelligence (AI) can enhance the reliability and intelligence applications tremendously. A major challenge of applying AI technique in BioMEMS is the lack of a universal algorithm. Most AI applied in BioMEMS is highly application-specific and cannot be easily used to other areas. One of the potential solutions is to create a BioMEMS system or platform in a hierarchy way and isolate each AI content into multiple-choice problems to be determined by users.

This Research Topic demonstrates that bio-mimicking, a basic principle in synthetic biology, can be an effective way to develop novel designs in BioMEMS. With the assist of BioMEMS, scientists have brought synthetic biology into a new era. The advanced BioMEMS synthetic biology includes using cells to conduct engineering problems, and using engineering products to mimic and solve biological problems that cannot be addressed by traditional biological strategies.

The four papers included in this Research Topic are well presented with contents, technologies, and the applications in multidisciplinary. The advanced BioMEMS technologies in microfluidics has a great potential to improve the performanceof traditional lab-on-chip (LOC), point-of-care (POC), organ-on-chip, programmable microfluidics, and recently developed lab-at-home (L@H) techniques (advanced BioMEMS technologies compatible with smart devices for daily usage at home). Advanced microfluidics and BioMEMS provide diversified approaches for developing and integrating sensors/transducers into microsystems.

